# Case report: Kikuchi-Fujimoto disease presenting with persistent fever and widespread lymphadenopathy in a young adult

**DOI:** 10.3389/fimmu.2024.1519988

**Published:** 2025-01-10

**Authors:** Jing Ye, Qian Yu, Yan Chen, Chunping Huang

**Affiliations:** ^1^ Department of General Practice, The Affiliated Panyu Central Hospital, Guangzhou Medical University, Guangzhou, China; ^2^ Department of Pathology, The Affiliated Panyu Central Hospital, Guangzhou Medical University, Guangzhou, China

**Keywords:** Kikuchi-Fujimoto disease, lymphadenopathy, viral infections, autoimmune, differential diagnosis

## Abstract

Kikuchi-Fujimoto disease (KFD) is a rare, self-limiting condition typically characterized by fever and lymphadenopathy. The exact etiology remains unclear but is suspected to be associated with viral infections and autoimmune responses. This report presents the case of a 32-year-old Chinese male who was admitted with recurrent high fever, lymphadenopathy, and hepatosplenomegaly. Initial treatment was ineffective, and a lymph node biopsy subsequently confirmed the diagnosis of KFD, with evidence of cytomegalovirus infection. Following treatment with corticosteroids, the patient’s symptoms improved rapidly, and no relapse was observed during follow-up after discharge. This case highlights the diagnostic challenges of KFD, particularly in distinguishing it from lymphoma and systemic lupus erythematosus. Accurate and timely diagnosis is crucial to avoid unnecessary treatments, and long-term follow-up is recommended to monitor for potential disease progression.

## 1 Introduction

Kikuchi-Fujimoto disease (KFD), also known as Kikuchi disease or histiocytic necrotizing lymphadenitis, is a rare disease of unknown etiology first reported in Japan in 1972. While it predominantly affects Asian populations, cases have been reported worldwide (1, 2). KFD can occur at any age, including in young children, but it most commonly affects adults under 40 years old (3–5). The main clinical manifestations are persistent fever and cervical lymphadenopathy, with other common symptoms including rash, nausea, vomiting, fatigue, arthralgia, and, in some cases, hepatosplenomegaly (6–8). The exact cause of KFD remains unclear, but viral infections and autoimmune mechanisms are considered potential triggers (2). Diagnosing KFD is challenging, with lymph node biopsy being crucial for confirmation. Up to 40% of cases are reportedly misdiagnosed, frequently mistaken for lymphoma, systemic lupus erythematosus (SLE), or even tuberculosis (9). Given that KFD is a relatively self-limiting disease, timely and accurate diagnosis is essential to avoid unnecessary treatments.

We report a case of a young Chinese male diagnosed with KFD, presenting with persistent fever, lymphadenopathy, and hepatosplenomegaly. The diagnosis was confirmed through lymph node biopsy, and remission was achieved following steroid treatment. This case highlights the clinical and pathological features of KFD and underscores the importance of differential diagnosis to distinguish KFD from conditions such as lymphoma and SLE.

## 2 Case report

A 32-year-old male was admitted to the hospital due to recurrent high fever and lymphadenopathy. Approximately one week prior to admission, the patient had been in good health before developing an unexplained high fever, with temperatures peaking at 40°C, accompanied by chills, fatigue, and poor appetite. He self-medicated with ibuprofen, but his condition did not improve. He was then evaluated in our outpatient clinic. The patient had no significant medical history, was not taking any other medications, and had no known allergies. There were no notable events in the six months preceding the onset of symptoms. He did not smoke or drink alcohol, and there was no relevant family history. Physical examination at the outpatient clinic revealed bilateral cervical, axillary, and inguinal lymphadenopathy, with firm, well-defined, painful lymph nodes, while the remainder of the physical exam was unremarkable. Laboratory tests showed elevated C-reactive protein (CRP) at 26.50 mg/L (0-10.0 mg/L) and leukopenia with a white blood cell count of 2.75×10^9^/L (3.5-9.5×10^9^/L). Tests for mycoplasma pneumoniae, influenza virus, and dengue fever were negative, and a chest CT scan was unremarkable. After initial antipyretic treatment failed to improve his symptoms, the patient was admitted for further investigation.

Upon admission, his body temperature was 38.1°C, blood pressure was 96/63 mmHg, pulse rate was 102 beats per minute, and respiratory rate was 18 breaths per minute. Physical examination revealed multiple enlarged lymph nodes in the cervical, axillary, and inguinal regions, with the largest node in the right cervical area. The lymph nodes were firm, well-defined, and painful, and hepatosplenomegaly was also detected. Other physical findings were unremarkable. Repeat laboratory tests revealed a white blood cell count of 1.46×10^9^/L (3.5-9.5×10^9^/L), CRP of 50.8 mg/L (0-10.0 mg/L), erythrocyte sedimentation rate (ESR) of 33 mm/h (3.5-9.5 mm/h), and positive cytomegalovirus (CMV) DNA. Other viral serologies, tests for acid-fast bacilli, antinuclear antibodies (ANA), and anti-double-stranded DNA antibodies were negative. **Table 1** summarizes the patient’s laboratory results, along with reference ranges. Ultrasound of the lymph nodes revealed multiple enlarged lymph nodes in the cervical, axillary, and inguinal regions. Based on the patient’s history and findings, hematologic diseases, immune system disorders, and KFD were considered as potential diagnoses.

**Table 1:** Laboratory data.

Variables	Pre-admission	On admission	Prior-discharge	Reference range
White blood cell count (×10^9^/L)	2.75	1.46	4.46	3.5–9.5
Neutrophil (×10^9^/L)	1.58	1.01	3.45	1.8–6.3
Lymphocyte (×10^9^/L)		0.29	0.71	1.1–3.2
Eosinophil (×10^9/^L)		0	0.01	0.02–0.52
C-reactive protein (mg/L)	26.5	50.8	13.38	0.0–10.0
Procalcitonin (ng/mL)		0.28		0.0–0.05
Red blood cell sedimentation rate (mm/h)		33		0.0–15
Interleukin-6 (pg/mL)		14.67		0.0–5.4
Interferon-γ (pg/mL)		741.17		0.0–23.1
Alanine aminotransferase, ALT (U/L)		19		9–50
Aspartate aminotransferase, AST (U/L)		43		15–40
Creatinine (μmol/L)		78		44–133
Rheumatologic and immunologic markers				
Rheumatoid factor (U/mL)		0.8		0–20
Complement factor 3 (g/L)		1.01		0.9–2.1
Complement factor 4 (g/L)		0.46		0.1–0.4
Anti-nuclear antibody titer		negative (-)		negative (-)
Anti-double-stranded DNA (IU/mL)		26.44		0–100
Immunoglobulin G (g/L)		9.9		7–16
Anti-Ro/SSA		negative (-)		negative (-)
Anti-La/SSB		negative (-)		negative (-)
Anti-Smith		negative (-)		negative (-)
Anti-cyclic peptide containing citrulline (U/mL)		<0.5		0.0–5
Pathogen Measurement				
CMV IgG (AU/mL)		80.8		<6
CMV IgM (S/CO)		0.19		<0.85
CMV DNA (copies/mL)		13000		<1000
Tuberculin intradermal reaction		negative (-)		negative (-)
Sputum smear (for acid-fast bacilli)		negative (-)		negative (-)
HSV-1/HSV-2 IgG		negative (-)		negative (-)
HBsAg (IU/mL)		negative (-)		negative (-)

CMV, cytomegalovirus; HSV, human herpes simplex virus.

**Figure 1:**
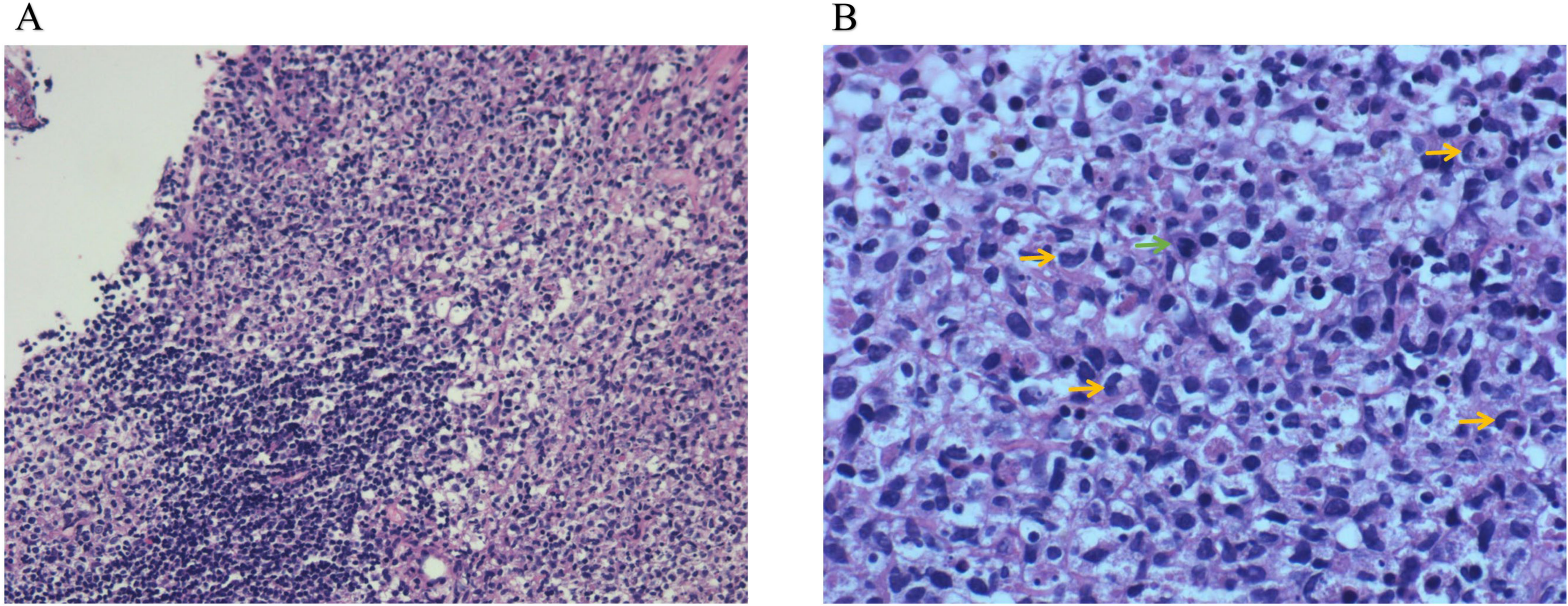
Lymph node biopsy pathology. **(A)** HE (low-power lens), **(B)** HE (high-power lens). Lymph node staining revealed features consistent with histiocytic necrotizing lymphadenitis. There was hyperplasia of the paracortical region, with prominent karyorrhexis in necrotic areas. Peripheral cells were larger, with pale-staining nuclei, and some cells displayed crescent-shaped nuclei (yellow arrows) and nuclear division (green arrows).

**Figure 2:**
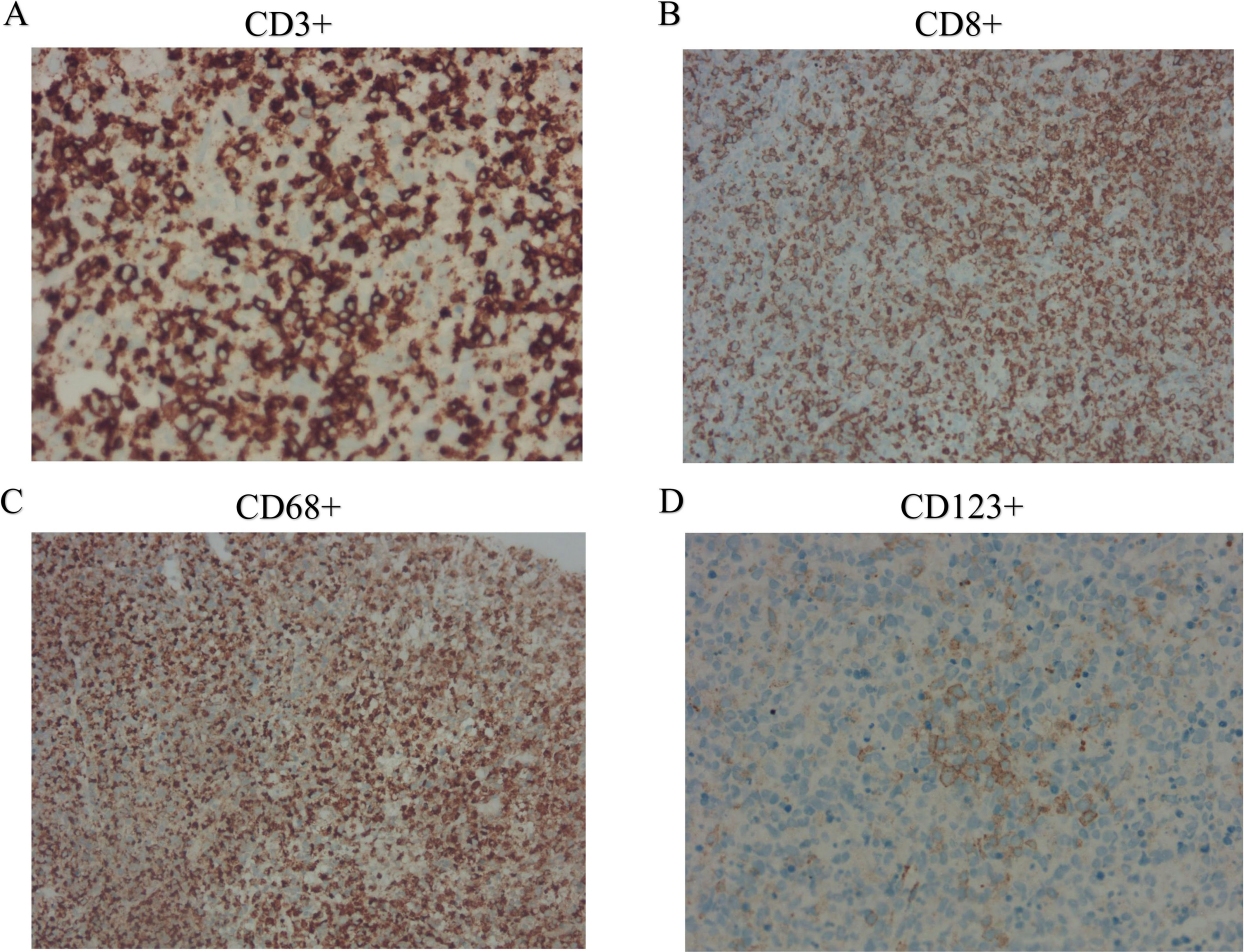
Immunohistochemical findings of cervical lymph nodes. **(A)** CD3+ staining demonstrates abundant T cells in the lymph node paracortex; **(B)** CD8+ staining highlights the predominance of cytotoxic T cells; **(C)** CD68+ staining reveals an abundance of histiocytes; and **(D)** CD123+ staining indicates the presence of plasmacytoid dendritic cells.

**Figure 3:**
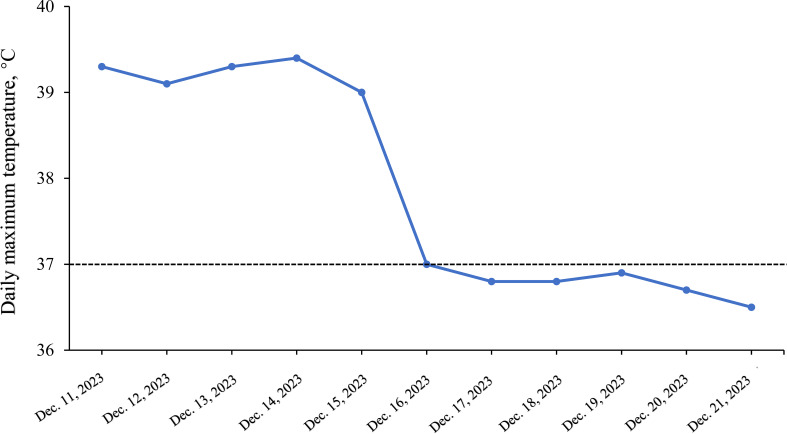
Daily maximum temperature variations during hospitalization.

Further evaluations, including PET-CT, bone marrow aspiration, and lymph node biopsy, were performed. PET-CT revealed multiple enlarged lymph nodes with increased glucose metabolism, hepatosplenomegaly, and diffusely increased glucose metabolism in axial bone marrow, suggesting necrotizing lymphadenitis. Bone marrow aspiration showed hypoplasia with 1.5% atypical lymphocytes, and peripheral blood revealed 3.0% atypical lymphocytes and leukopenia. Lymph node biopsy demonstrated hyperplasia of the paracortical area, predominantly of cytotoxic T cells, with numerous irregular and crescent-shaped histiocytes and proliferating plasmacytoid dendritic cells, along with extensive nuclear debris and areas of patchy necrosis, which are hallmark features of KFD (**Figure 1**). Immunohistochemical staining revealed an abundance of CD3+ T cells, with a predominance of CD8+ cells over CD4+ cells, indicating a cytotoxic T-cell-dominant response. CD123 staining highlighted the presence of plasmacytoid dendritic cells. CD20 staining showed B cells predominantly localized to uninvolved areas, with an absence in karyorrhectic regions. MPO staining was positive in foamy and crescent-shaped histiocytes. Additionally, Ki-67 positivity (~70%) and CD68 positivity were observed, while molecular pathology demonstrated EBER-ISH negativity (**Figure 2**). Based on these findings, a definitive diagnosis of KFD was made. Following the diagnosis of KFD, the patient was treated with intravenous methylprednisolone at 40 mg/day. The patient’s general condition improved after steroid therapy, and his fever resolved on the second day of treatment (**Figure 3**). The pain in the lymph nodes subsided, and by the third day, physical examination revealed a marked reduction in lymph node size. After one week of steroid therapy, the patient was discharged with significant symptom improvement. One month later, during a follow-up visit, the patient had no fever, and physical examination revealed no lymphadenopathy in the cervical, axillary, or inguinal regions. Follow-up ultrasound of the lymph nodes and abdomen showed no abnormal enlargement of lymph nodes, and no hepatosplenomegaly was observed. The patient has now been followed for 10 months with no signs of recurrence. However, continuous follow-up is necessary to monitor for potential recurrence or the development of SLE or other autoimmune diseases.

## 3 Discussion

KFD typically presents with an acute or subacute onset, characterized by fever and lymphadenopathy, with lymph node enlargement predominantly localized to the cervical region. However, a recent study found that lymph node involvement in other body regions occurs in up to 23.9% of cases, suggesting that generalized lymphadenopathy is also common (10). Currently, there is no consistent laboratory finding associated with KFD, though many cases have been reported with leukopenia, anemia, and elevated erythrocyte sedimentation rate (11). KFD is a self-limiting inflammatory disorder with an unclear etiology (1). The pathogenesis of the disease is generally believed to involve two main mechanisms: viral infection and autoimmune response (2). Leukopenia is thought to be mediated by cytokine-induced mechanisms. It has been reported that up to 25% of patients have atypical lymphocytes in peripheral blood, supporting the proposed viral etiology of the disease (11, 12). Dorfman et al. also observed positive viral serology in some KFD patients (13), with common viruses including Epstein-Barr virus (EBV), CMV, and parvovirus. The inflammatory response triggered by viral infection plays a crucial role in the antiviral process, involving the activation and proliferation of CD8+ T cells, CD4+ T cells, and dendritic cells (14). As a result, viral infections disrupt the immune homeostasis of the body, leading to increased release of inflammatory cytokines (15). Electron microscopy has revealed the presence of tubular reticular structures within the cytoplasm of stimulated lymphocytes and histiocytes in individuals diagnosed with KFD (16). Therefore, KFD is hypothesized to be a self-limiting autoimmune condition triggered by viral-infected lymphocytes (17, 18).

Accurate diagnosis of KFD requires histopathological examination of the affected lymph nodes. The characteristic pathological features of KFD include partial destruction of lymph node architecture, with focal necrosis in the cortical and paracortical areas, accompanied by abundant nuclear debris. These necrotic areas are surrounded by significant histiocytic infiltration, which includes immunoblasts and lymphocytes. The histiocytes exhibit varying morphologies, including crescent-shaped, phagocytic, and foamy histiocytes. Based on these pathological features, the affected lymph nodes can be classified into three types: proliferative, necrotizing, and xanthomatous (12). The proliferative type represents the early stage of the disease, characterized by enlargement of the paracortical areas, with an increase in histiocytes and plasmacytoid dendritic cells, mixed with lymphocytes and nuclear debris. Once necrosis is present, the case is classified as necrotizing. In the xanthomatous type, foamy histiocytes dominate the lesion, regardless of the presence of necrosis (11, 12).

The patient in this case presented with acute onset of fever and lymphadenopathy. The pathological results of the lymph node biopsy revealed paracortical hyperplasia, predominantly involving cytotoxic T-cell proliferation, along with numerous irregular and crescent-shaped histiocytes, plasmacytoid dendritic cell proliferation, and widespread nuclear debris. These histological features are most consistent with a diagnosis of KFD. However, differential diagnoses should include lymphoma, SLE, and infectious lymphadenopathy.

KFD is often misdiagnosed as T-cell lymphoma, which poses a significant pitfall for clinicians. T-cell lymphoma typically occurs in patients over 50 years of age, and in our case, further immunohistochemical staining showed MPO positivity, with molecular pathology indicating EBER-ISH negativity, supporting the exclusion of T-cell lymphoma. The abundant presence of crescent-shaped histiocytes is also a hallmark of KFD rather than lymphoma. In KFD patients, compared to other non-neoplastic necrotizing lymphadenitis, lymph nodes usually lack neutrophils and eosinophils, but show abundant crescent-shaped histiocytes.

Differentiating KFD from SLE is particularly challenging due to clinical and pathophysiological similarities. Lymph nodes from patients with SLE-associated lymphadenitis typically contain more neutrophils and plasma cells than those from KFD patients, and vasculitis outside necrotic areas is more common in SLE. However, this patient did not display these features. Nonetheless, serological testing for SLE is recommended in patients with biopsy findings suggestive of KFD, as histological features alone may not always clearly distinguish the two. In this case, all SLE-related serological tests were negative, and the patient did not exhibit facial rash, joint pain, lupus nephritis, or neuropsychiatric involvement, which are characteristic of SLE. The relationship between KFD and SLE can manifest in several ways: (1) KFD may precede the onset of SLE; (2) KFD and SLE may coexist; (3) KFD may evolve into SLE (19, 20). Since KFD is also associated with other autoimmune diseases, such as antiphospholipid syndrome and rheumatoid arthritis, and not exclusively SLE, KFD and SLE should not be viewed as mutually exclusive diagnoses, as doing so could lead to underdiagnosis of KFD. This means that even in the presence of antinuclear antibodies, a diagnosis of KFD remains valid as long as the typical histological features are present in the lymph nodes. These patients should be followed long-term to monitor for the development of SLE. A few case reports have documented patients being diagnosed with SLE several months or even years after lymph node biopsy findings suggested KFD, indicating that some patients initially diagnosed with KFD may actually be in the early stages of SLE. Of course, the likelihood of developing SLE is lower in patients with no abnormality in SLE-specific antibodies at the time of a confirmed KFD diagnosis (11).

KFD often presents a diagnostic challenge by mimicking malignancies such as lymphoma or autoimmune diseases like SLE. In this case, generalized lymphadenopathy and hepatosplenomegaly initially raised concerns for malignancy, prompting extensive testing. However, the rapid onset of tender lymph nodes and benign histopathological features ultimately revealed KFD. This underscores the critical importance of tissue biopsy in distinguishing KFD from more ominous diagnoses and resolving diagnostic dilemmas where clinical and imaging findings overlap, ultimately sparing patients from unnecessary treatments and interventions.

Notably, the case highlights the importance of considering KFD in male patients, a group less frequently associated with the condition. Early recognition not only prevents diagnostic delays but also alleviates the significant psychological burden that comes with suspected malignancy, particularly in younger patients. Furthermore, timely diagnosis facilitates long-term follow-up to monitor for autoimmune conditions such as SLE, which may emerge over time. This case serves as a reminder that rare diseases like KFD can hide in plain sight, challenging clinicians to maintain a broad differential diagnosis and rely on meticulous pathological evaluation to navigate diagnostic uncertainty.

The patient’s symptoms improved rapidly following corticosteroid therapy. At follow-up after discharge, the patient’s symptoms had resolved, lymphadenopathy had nearly disappeared, blood counts had returned to normal, and the patient was feeling well and had returned to work. The patient continues to be monitored through ongoing follow-up to track his condition.

## Data Availability

The original contributions presented in the study are included in the article/**Supplementary Material**. Further inquiries can be directed to the corresponding author.

## Supplementary material

Supplementary Figure 1 Timeline of disease diagnosis and treatment.

## References

[1] Cuglievan B Miranda RN. Kikuchi-fujimoto disease. Blood. (2017) 129:917. doi: 10.1182/blood-2016-08-736413 28209753

[2] Perry AM Choi SM. Kikuchi-fujimoto disease: A review. Arch Pathol Lab Med. (2018) 142:1341–6. doi: 10.5858/arpa.2018-0219-RA 30407860

[3] Chuang CH Yan DC Chiu CH Huang YC Lin PY Chen CJ. Clinical and laboratory manifestations of Kikuchi’s disease in children and differences between patients with and without prolonged fever. Pediatr Infect Dis J. (2005) 24:551–4. doi: 10.1097/01.inf.0000167246.24500.21 15933568

[4] Kang HM Kim JY Choi EH Lee HJ Yun KW Lee H. Clinical characteristics of severe histiocytic necrotizing lymphadenitis (Kikuchi-fujimoto disease) in children. J Pediatrics. (2016) 171:208–212.e201. doi: 10.1016/j.jpeds.2015.12.064 26852178

[5] Lin YC Huang HH Nong BR Liu PY Chen YY Huang YF. Pediatric Kikuchi-Fujimoto disease: A clinicopathologic study and the therapeutic effects of hydroxychloroquine. J Microbiol Immunol Infection Wei Mian Yu Gan Ran Za Zhi. (2019) 52:395–401. doi: 10.1016/j.jmii.2017.08.023 29050748

[6] Huang J Zheng JX Yang Y Zhu D. Necrotizing lymphadenitis: A case report and literature review. Z Fur Rheumatol. (2021) 80:274–82. doi: 10.1007/s00393-020-00929-6 33241524

[7] Tan HM Hue SS Wee A See KC. Kikuchi-fujimoto disease post COVID-19 vaccination: case report and review of literature. Vaccines. (2021) 9(11):1251. doi: 10.3390/vaccines9111251 PMC8624158 34835182

[8] Park S Kim JY Ryu YJ Lee H. Kikuchi cervical lymphadenitis in children: ultrasound differentiation from common infectious lymphadenitis. J Ultrasound Med: Off J Am Institute Ultrasound Med. (2021) 40:2069–78. doi: 10.1002/jum.v40.10 33263358

[9] Ramirez AL Johnson J Murr AH. Kikuchi-Fujimoto’s disease: an easily misdiagnosed clinical entity. Otolaryngol–head Neck Surgery: Off J Am Acad Otolaryngol-Head Neck Surgery. (2001) 125:651–3. doi: 10.1067/mhn.2001.120431 11743471

[10] Razak AA Shanmugasundaram S. Kikuchi-Fujimoto disease, a rare benign disease with atypical histomorphology: more than meets the eye. Pathology. (2024) 56:382–90. doi: 10.1016/j.pathol.2023.10.017 38296677

[11] Kucukardali Y Solmazgul E Kunter E Oncul O Yildirim S Kaplan M. Kikuchi-Fujimoto Disease: analysis of 244 cases. Clin Rheumatol. (2007) 26:50–4. doi: 10.1007/s10067-006-0230-5 16538388

[12] Bosch X Guilabert A Miquel R Campo E. Enigmatic Kikuchi-Fujimoto disease: a comprehensive review. Am J Clin Pathol. (2004) 122:141–52. doi: 10.1309/YF081L4TKYWVYVPQ 15272543

[13] Dorfman RF Berry GJ. Kikuchi’s histiocytic necrotizing lymphadenitis: an analysis of 108 cases with emphasis on differential diagnosis. Semin Diagn Pathol. (1988) 5:329–45. 3217625

[14] Asano S Mori K Yamazaki K Sata T Kurata A Sato Y. Necrotizing lymphadenitis (NEL) is a systemic disease characterized by blastic transformation of CD8+ cells and apoptosis of CD4+ cells. Virchows Archiv: An Int J Pathol. (2014) 464:95–103. doi: 10.1007/s00428-013-1516-z 24292234

[15] Sun L Su Y Jiao A Wang X Zhang B. T cells in health and disease. Signal Transduction Targeted Ther. (2023) 8:235. doi: 10.1038/s41392-023-01471-y PMC10277291 37332039

[16] Rosado FG Tang YW Hasserjian RP McClain CM Wang B Mosse CA. Kikuchi-Fujimoto lymphadenitis: role of parvovirus B-19, Epstein-Barr virus, human herpesvirus 6, and human herpesvirus 8. Hum Pathol. (2013) 44:255–9. doi: 10.1016/j.humpath.2012.05.016 22939574

[17] Bosch X Guilabert A. Kikuchi-fujimoto disease. Orphanet J Rare Dis. (2006) 1:18. doi: 10.1186/1750-1172-1-18 16722618 PMC1481509

[18] Rönnblom L Alm GV. A pivotal role for the natural interferon alpha-producing cells (plasmacytoid dendritic cells) in the pathogenesis of lupus. J Exp Med. (2001) 194:F59–63. doi: 10.1084/jem.194.12.f59 PMC2193578 11748288

[19] Patra A Bhattacharya SK. SLE developing in a follow-up patient of kikuchi’s disease: A rare disorder. J Clin Diagn Res: JCDR. (2013) 7:752–3. doi: 10.7860/JCDR/2013/5017.2904 PMC3644467 23730669

[20] Kim SK Kang MS Yoon BY Kim DY Cho SK Bae SC. Histiocytic necrotizing lymphadenitis in the context of systemic lupus erythematosus (SLE): Is histiocytic necrotizing lymphadenitis in SLE associated with skin lesions? Lupus. (2011) 20:809–19. doi: 10.1177/0961203310397684 21562017

